# The interplay of sleep disorders and orofacial pain: A systematic review

**DOI:** 10.4317/jced.63216

**Published:** 2025-10-17

**Authors:** Pascual Colonques-Sanmartín, Maria Margaix-Muñoz, Leticia Bagán

**Affiliations:** 1 Graduated in Dentistry. University of Valencia. Valencia, Spain; 2 Lecturer. Oral Medicine Unit. University of Valencia. Valencia, Spain; 3 Senior Lecturer. Oral Medicine Unit. University of Valencia. Valencia, Spain

## Abstract

**Background:**

Chronic orofacial pain (OFP) and sleep disorders are highly prevalent conditions that significantly impact quality of life. Increasing evidence suggests a bidirectional relationship between these disorders, whereby sleep disturbances may exacerbate OFP and vice versa. This systematic review synthesizes current evidence on the influence of sleep disorders on chronic OFP and explores additional factors that may contribute to this interaction.

**Material and Methods:**

A systematic review was conducted following PRISMA 2020 guidelines and registered the study with PROSPERO (CRD4202525111587). PubMed, Scopus, and Web of Science were searched for relevant literature published between 2004 and March 2024. Randomized controlled trials, cross-sectional studies, case-control studies, and cohort studies examining the association between sleep disorders and OFP were included. Ten studies met the eligibility criteria.

**Results:**

A consistent association was identified between chronic OFP and sleep disorders, particularly insomnia and obstructive sleep apnea (OSA), with stronger correlations observed in women. Limited evidence also suggests that OFP severity may increase with age and that greater sleep disturbance correlates with more intense pain. Limitations: The paucity of high-quality studies specifically addressing the relationship between sleep disorders and chronic orofacial pain was a notable issue. Additionally, there was significant methodological heterogeneity among the included studies, especially regarding study design, diagnostic criteria, and assessment tools. The decision to include only studies focused on orofacial pain and to exclude other categories of pain-related diseases, such as temporomandibular joint disorders (TMD), introduced an additional challenge and resulted in a reduction of the number of selected studies.

**Conclusions:**

There is a strong connection between chronic orofacial pain (OFP) and sleep disorders, particularly insomnia and obstructive sleep apnea (OSA). Addressing sleep-related issues could be an effective complementary approach in managing OFP, especially in female patients. More research is necessary to clarify the underlying mechanisms and to develop targeted, interdisciplinary interventions.

## Introduction

Sleep, a fundamental and universally conserved biological process, occupies approximately one-third of human life (1). Characterized by a state of relative quiescence and reduced responsiveness to external stimuli, sleep is essential for various physiological functions. It interacts bidirectionally with multiple bodily systems, influencing processes from immune function to cognitive performance (2). Orofacial pain (OFP) refers to a variety of pain conditions that are localized to the face and oral cavity. This pain can originate from nearby structures, result from nervous system dysfunction, or be referred from distant sources (3). OFP includes various types of pain such as dental, mucosal, musculoskeletal, neurovascular, and neuropathic pain (4). Researchers estimate that the prevalence of OFP in the general U.S. population ranges from 10% to 25% (3). Chronic pain is defined as pain that persists or recurs for more than three months (5). This type of pain continues beyond the expected healing period and may remain even after the initial cause has resolved. Chronic orofacial pain is a complex condition that involves biological, psychological, and social factors, all of which can significantly impair daily functioning, emotional well-being, and overall quality of life (6). Additionally, orofacial musculoskeletal pain disrupts the body's homeostatic balance, interferes with goal-directed behaviour, and generates stress. Conversely, psychosocial burdens can negatively affect the central nervous system and contribute to neuropsychological issues, insomnia, musculoskeletal pain, and autonomic nervous system dysregulation (7). Recent investigations have shown a significant interaction between sleep quality, fatigue, and OFP subtypes. Evidence from studies has demonstrated a strong association between pain severity, pain interference, and sleep problems (8). Sleep disorders, defined as medical conditions that disrupt restful sleep, can lead to daytime sleepiness and dysfunction. The International Classification of Sleep Disorders (ICSD) and the American Academy of Sleep Medicine provide widely used classification systems (9). The ICSD, in its third edition, categorizes sleep disorders into seven main categories: insomnia, sleep-disordered breathing, central disorders of hypersomnolence, circadian rhythm sleep-wake disorders, parasomnias, sleep-related movement disorders, and other sleep disorders (10). This review focuses on the most prevalent sleep disorders: insomnia, obstructive sleep apnea (OSA), and sleep bruxism (SB) (11). Disturbances in the initiation and continuity of sleep, or experiencing early morning awakenings, characterizes insomnia (12). OSA involves recurrent upper airway obstruction leading to blood oxygen desaturation and nocturnal arousals (13). Sleep bruxism, characterised as rhythmic (phasic) or non-rhythmic (tonic) masticatory muscle activity during sleep, can cause arousals and sleep fragmentation (14). Polysomnography is the most reliable method for SB diagnosis (13). Sleep disorders and chronic orofacial pain represent two significant health concerns that affect a considerable proportion of the worldwide population. Sleep disorders, including insomnia, sleep bruxism, and sleep apnea, are highly prevalent and can exert a substantial impact on patients. Similarly, chronic orofacial pain, akin to sleep disorders, also has a considerable influence on a significant number of individuals. The coexistence of these two entities has been a subject of increasing interest in recent years in sleep medicine and dentistry, with clinical studies suggesting a connection between the two of them. Given the increasing incidence of these diseases and their current significance, the primary objective of this review was to analyse the influence of sleep disorders on chronic orofacial pain. As supplementary objectives, other pertinent aspects were taken into consideration, including the identification of the sleep disorder with the greatest influence on chronic orofacial pain, the analysis of the origin of this influence, if it is chronic orofacial pain or sleep disorders, and the determination of whether the severity of sleep disorders is associated with the severity of orofacial pain.

## Material and Methods

This systematic review was conducted following the PRISMA 2020 guidelines (15) and registered the study with PROSPERO (CRD4202525111587). The PICO (Population, Intervention, Comparison, Outcome) framework was used to formulate the research question: Is there an influence of sleep disorders on orofacial pain? P (Population): Individuals with sleep disorders and chronic orofacial pain. I (Intervention): Assessment of sleep disorders and/or orofacial pain. C (Comparison): Individuals without sleep disorders or orofacial pain. O (Outcome): Assessing the influence of sleep disorders on orofacial pain. Relevant studies were included: randomised controlled clinical trials, cross-sectional, case-control, and cohort studies (both prospective and retrospective), while excluding case studies, meta-analyses, systematic reviews, literature reviews, animal studies, and in vitro studies. Regarding articles concerning sleep bruxism and other diseases that could be associated with the spectrum of temporomandibular joint diseases were excluded from our review it is essential to note that only those on orofacial pain were the sole focus of our rigorous review. A meticulous search strategy was employed across three major databases: PubMed, Scopus, and Web of Science. The literature search was finished in March 2024 (Table 1).

Table 1

**Figure 1:**
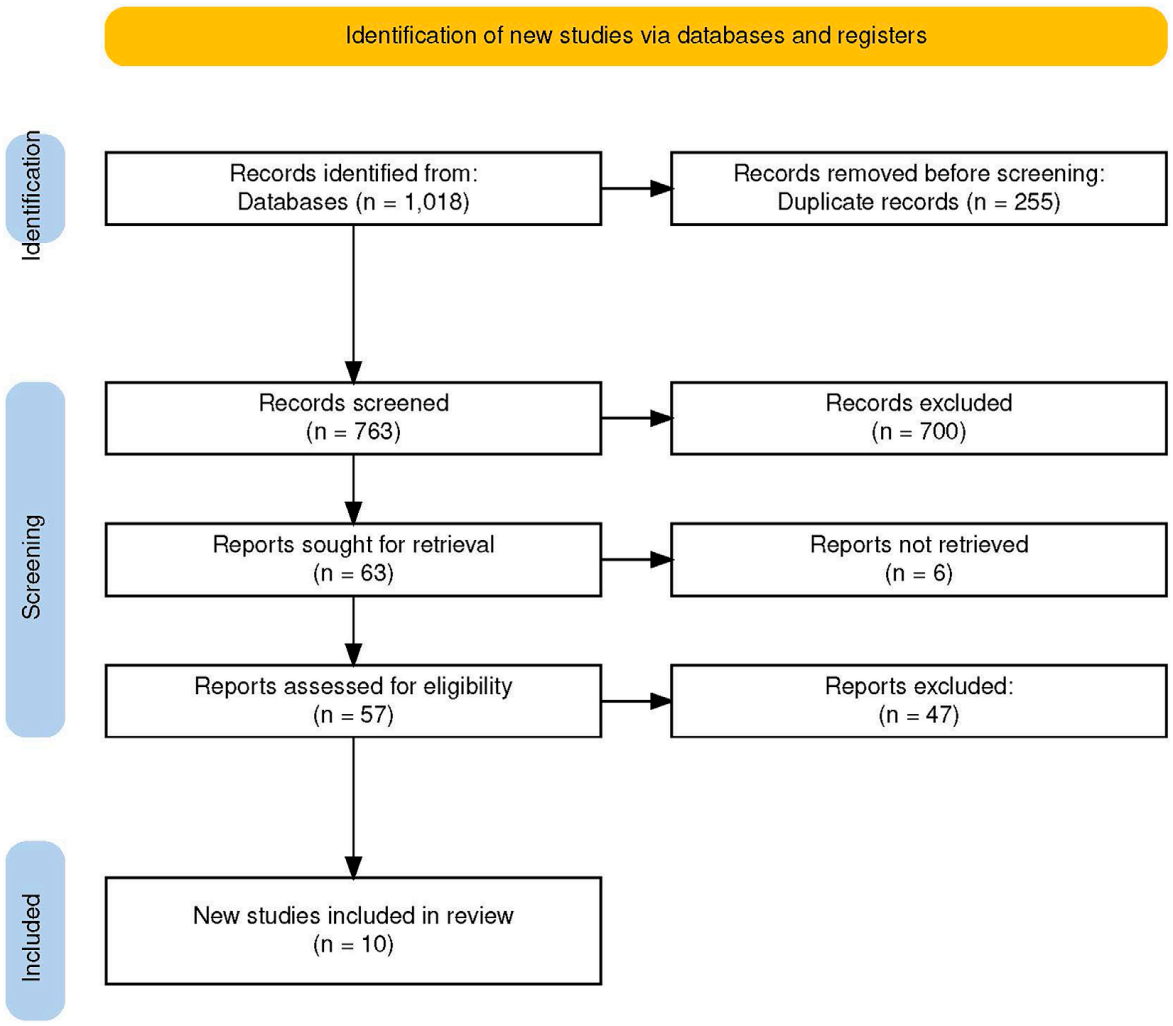
PRISMA flow diagram of study selection. PRISMA: Preferred Reporting Items for Systematic Reviews and Meta-Analyses.

Following the removal of duplicates (n=255), a total of 763 articles were screened based on title and abstract, leading to the identification of 66 articles that met the criteria for full-text eligibility assessment. Following the removal of 47 articles deemed to be irrelevant to orofacial pain, a total of 10 articles were selected for inclusion in the systematic review (Fig. 1, Table 2).

1

Table 2

The search and retrieval of articles was independently performed by two reviewers (PC, MM).

## Results

**Figure 2:**
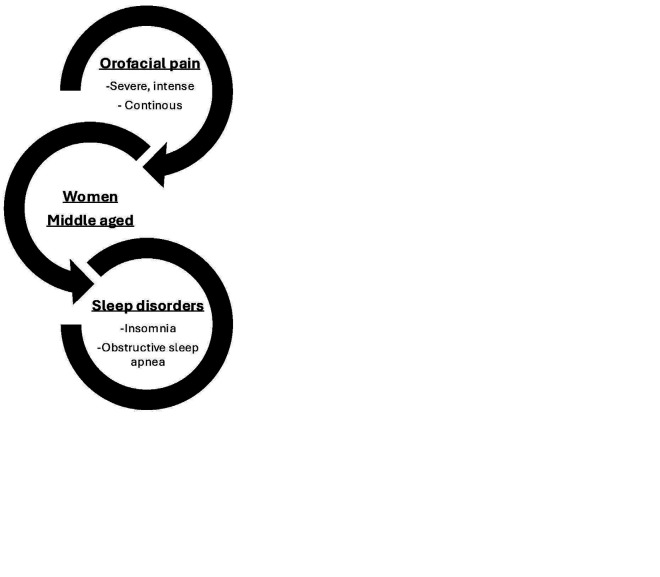
A graphic illustration showing the main results and how they are interrelated.

The majority of reviewed studies demonstrate a significant association between chronic orofacial pain and sleep disorders, particularly insomnia and OSA. However, there are exceptions to this association, including the study by Kang et al. (16), which found no significant relationship between obstructive sleep apnea and chronic orofacial pain but did identify a significant relationship between insomnia and orofacial pain. Conversely, Ning et al. (17) observed that patients with OSA can develop orofacial pain. The study revealed significant sex differences, showing a stronger association between orofacial pain and sleep disorders in women, which is consistent with the higher prevalence of orofacial pain in the female population. Camparis et al. (18) showed a 7.7:1 ratio in the prevalence of orofacial pain in patients with sleep bruxism between women and men. Some authors agreed that the intensity of pain related to sleep disorders increases with age. However, Iturriaga et al. (19) did not find statistically significant results in this relationship. Different studies have investigated the severity of sleep disorders, which vary by the type of sleep problem each patient presents. Both the intensity of pain and its functional impact are independently associated with sleep problems, with the latter being the strongest correlate (8). The psychosocial morbidity of orofacial pain is widely recognized among the studies reviewed (Fig. 2).

2

Regarding the diagnostic methods employed in the studies about orofacial pain, the vast majority used orofacial pain questionnaires such as the VAS questionnaire or the brief orofacial pain questionnaire as an assessment instrument. Furthermore, several studies incorporated physical examinations of the following nature: facial muscle palpation and identification of dental status through a panoramic X-ray. These served the purpose of diagnostic methods for orofacial pain (18). Then, to assess sleep quality, certain studies, such as Lee et al. (20) or Ravindranath et al. (8), used the Pittsburgh Sleep Quality Index. As for OSA, the most commonly used diagnostic method was the STOP-BANG questionnaire, which determines the risk of obstructive apnea, as in the articles by Ning et al. (17) and Kang et al. (16).

## Discussion

This systematic review investigated the relationship between sleep disorders and chronic orofacial pain. Our analysis of ten studies shows a significant association between these conditions, consistent with a growing body of evidence suggesting a bidirectional relationship. The literature reviewed indicates that sleep disturbance is more common in patients with chronic OFP than in the general population, supporting the notion that sleep disturbance may exacerbate pain and vice versa (21 , 22).

Thus, the results are consistent with previous studies reporting an association between sleep disturbance and orofacial pain. Benoliel et al. (23), Porto et al. (24), Kim et al. (25), and Schmitter et al. (26) concluded that a considerable number of patients (approximately 20%) with orofacial pain reported being aware of awakenings or poor sleep quality. Continuous pain was the most significant parameter associated with awakening; persistent orofacial pain often induced pain-related awakening, and that was significantly associated with pain intensity (23). Furthermore, and reciprocally, according to Sivertsen et al. (27), insomnia and poor sleep quality may lead to decreased pain tolerance in patients with chronic orofacial pain, thus increasing the sensation of discomfort in the facial region and, per se, explaining the interaction of sleep disturbances in orofacial pain.

Regarding obstructive sleep apnea, Smith et al. (28) reported that 28% of patients with orofacial pain have OSA. Similar to insomnia and OSA, sleep bruxism has been linked to orofacial pain in multiple studies. Other research suggests that sleep bruxism also affects sleep disorders like OSA and insomnia, creating a vicious cycle that worsens these conditions and increases orofacial pain. This indicates that bruxism may lead to sleep disorders such as parasomnias and insomnia (29 , 30). Conversely, De Luca Canto et al. (31), in their systematic review, argued that sleep bruxism does not impact other sleep disorders or orofacial pain.

Upon examining the current literature concerning the influence of different factors on sleep disorders and orofacial pain, it has been observed that gender, specifically female gender, shows an increased interactive impact. According to Haack et al. (36), sleep restriction and poor sleep quality lead to a greater likelihood of pain in women compared to men. Furthermore, Smith et al. (37) have demonstrated that sleep disturbances can diminish pain inhibitory function in women, potentially resulting in reduced pain tolerance. In addition to gender, it is interesting to consider race and ethnicity as feasible contributing factors to the interaction between pain and sleep disturbances. Hermans et al. (34) and Kim et al. (35) have postulated that racial and ethnic disparities exist with regard to experimental pain perception and orofacial pain modulation. These researchers acknowledged the well-documented variation in sleep patterns across different racial and ethnic groups.

It is hypothesised that sleep quality and pain modulation function deteriorate with age. This is evidenced by the heightened pain facilitation and diminished inhibition exhibited by older adults. Consequently, it is interesting to consider the influence of factors such as age, gender, and other variables, including race and ethnicity, on orofacial pain modulation in patients experiencing sleep disorders (36).

The findings of this study indicate that the association between sleep disorders and chronic orofacial pain can be explained by various biological mechanisms, in addition to shared pathophysiological mechanisms. This is due to the fact that orofacial pain and sleep disorders share common neurobiological pathways. Pain sensitization and serotonergic dysfunction have been identified by Lavigne et al. (36) as contributing factors to both chronic pain and sleep disturbances. Sleep disorders can induce chronic pain in patients with orofacial pain due to an inflammatory imbalance characterised by elevated IL-6 production. Patients with insomnia and poor sleep quality suffering from orofacial pain showed this IL-6 upregulation, thus demonstrating that the immune factor might play an important role in this interaction (37).

According to the reports from previous studies on this topic, sleep disorders and facial pain interact reciprocally. Poor sleep quality, insomnia, unrefreshing sleep, and problems in getting sleep act in a reciprocal and bidirectional manner with orofacial pain, although it is also pointed out that sleep disorders seem to be a more reliable predictor of pain onset and pain characteristics than the other way around, i.e., pain to sleep disorders (38). Patients with orofacial pain and cranio-mandibular dysfunction exhibit a cyclical relationship between a poor night's sleep and increased pain the following day, which may be attributed to mood disturbances, stress-related changes in the hypothalamic-pituitary-adrenal axis, or even genetic predisposition (37). A significant area for future research could be the clinical perspectives on the interrelationship between orofacial pain and sleep disorders. As previously stated, orofacial pain is closely related to some sleep disorders, and, conversely, sleep disorders such as apnea and insomnia may also contribute to the development and exacerbation of orofacial pain. This highlights the importance of understanding the bidirectional relationship of both pathologies for diagnosis and treatment in clinical practice. Similarly, patients with sleep disorders should be evaluated for signs of orofacial pain and other related craniofacial conditions. This underscores the necessity for a multidisciplinary and collaborative approach across diverse medical and dental specialties in the management of these conditions (39 , 40).

It is important to note that this systematic review has some limitations. First, there is a general scarcity of high-quality studies specifically addressing the relationship between sleep disorders and chronic orofacial pain, which limited the number of eligible articles despite a broad and structured search. Second, significant methodological heterogeneity was observed among the included studies, especially regarding study design, diagnostic criteria, and assessment tools. Some relied heavily on subjective questionnaires, while others provided limited details about clinical evaluation procedures. This variability hindered direct comparison. Importantly, the decision to exclude studies focused on temporomandibular joint disorders (TMD) created an additional challenge, as many publications in this field do not differentiate TMD from broader categories of orofacial pain. Consequently, several potentially relevant studies had to be discarded to maintain the review's specificity, further limiting the available data.

In conclusion, the present systematic review provides evidence of a strong association between sleep disorders, especially insomnia and obstructive sleep apnea, and chronic orofacial pain. Further investigation is necessary to elucidate the underlying mechanisms, determine potential moderating factors, and develop targeted interventions for patients with co-occurring sleep disorders and OFP.

## Figures and Tables

**Table 1:** Search equations employed in the databases consulted.

Database	Search equation
PubMed	((((facial pain[Title/Abstract]) OR (orofacial pain[Title/Abstract])) OR (chronic orofacial pain[Title/Abstract])) AND (((((sleep[Title/Abstract]) OR (sleep wake disorder[Title/Abstract])) OR (sleep disorder[Title/Abstract])) OR (sleep disturbance[Title/Abstract])) OR (sleep quality[Title/Abstract]))) NOT (rhinosinusitis[Title/Abstract])
Embase	“facial pain”:ab,ti OR “orofacial pain”:ab,ti OR “chronic orofacial pain”: ab,ti AND “sleep”:ab,ti OR “sleep wake disorder”:ab,ti OR “sleep disorder”:ab,ti OR “sleep disturbance”:ab,ti OR “sleep quality”:ab,ti NOT “rhinosinusitis”:ab,ti
Scopus	TITLE-ABS (facial AND pain) OR TITLE-ABS (orofacial AND pain) OR TITLE-ABS (chronic AND orofacial AND pain) AND TITLE-ABS (sleep) OR TITLE-ABS (sleep AND wake AND disorder) OR TITLE-ABS (sleep AND disorder) OR TITLE-ABS (sleep AND disturbance) OR TITLE-ABS (sleep AND quality) AND NOT TITLE-ABS (rhinosinusitis)

OSA: Obstructive Sleep Apnea; VAS: Visual Analogue Scale; OFP: Orofacial pain.

**Table 2:** Relevant data from the studies included in the systematic review.

Author, year and country	Type and design of the study	Sample size	Diagnostic method	Final diagnosis
Bonetti A. 2024. UK	Transverse	450 patients (82.2%) women Mean age 44.63 ± 15.98	Pain assessment questionnaires with the Graduated Scale of chronic and psychological pain. Clinical examination	Insomnia and chronic orofacial pain
Ning R. 2023. China.	Cohort	71 patients divided into 3 groups according to the intensity of OSA (45) Men (26) WomenMean age36.8± 3.5	Clinical examination of muscle palpation and pain questionnaire with a scale of ( 0 (no pain) 10 (severe pain) VAS. Assessing OSA severity with the STOP BANG questionnaire	OSA and chronic orofacial myofascial pain
Kang J. 2022. South Korea	Transverse	5780 patients (2503) men (3277) women Mean age 62± 12)	Questionnaires and physical examination of muscle palpation. VAS Questionnaire for Pain Assessment STOP BANG Questionnaire for OSA Risk	OSA, sleep quality, insomnia and chronic orofacial pain
Lee H. 2020. South Korea	Transverse	3276 patients (992) Men (2284) Women Mean age 34.82±16.78	Subjective Assessment of Pain Symptoms and Sleep Quality Questionnaire	Sleep awakenings, sleep quality, and chronic orofacial pain
Camparis CM. 2006. Brazil	Descriptive study	100 patients 2 groups: (70) with orofacial pain (30) No orofacial pain	VAS Orofacial Pain Questionnaire Cervical, cranial, facial, dental, and other structures evaluation	Bruxism and orofacial pain
Ahlberg K. 2005. Finland	Control cases	750 patients (78%) women Mean age 42.3 years	Standardized questionnaire containing in RDC/TMD Axis II. VAS Questionnaire for Orofacial Pain Assessment	Bruxism, insomnia, and orofacial pain
Ravindranath P.2023. UK	Cohort Study	460 patients (72.6%) women	Measuring Sleep Quality through the Chronic Pain Sleep Inventory and the Pittsburg Index Pain severity was measured with the Brief Orofacial Pain Inventory	Sleep disorders and orofacial pain
Meira e Cruz M. 2019. Switzerland	Transverse	952 patients (30.5%) men Mean age44.8 ± 17.4	Anonymized pain and sleep disorder questionnaires	Sleep disturbance, insomnia, daytime sleepiness and orofacial pain
Lee H. 2017. South Korea	Transverse	3279 patients 72.3% women Mean age 44.4	Pittsburgh Scale Questionnaire for Measuring Sleep Quality in Patients with Orofacial Pain	Insomnia, sleep duration, and orofacial pain
Iturriaga V. 2019 Chile	Transverse	225 patients 82.9% women	Sleep Quality and Daytime Sleepiness Questionnaire Evaluation of the degree of orofacial pain according to the VAS questionnaire.	Insomnia, sleep quality, daytime sleepiness, and orofacial pain

2

## Data Availability

The datasets used and/or analyzed during the current study are available from the corresponding author.
